# Impact on Hydrotherapy: Contribution by Sake Dean Mahomed

**DOI:** 10.6026/973206300211574

**Published:** 2025-06-30

**Authors:** Mandala Sathwik, Satya Lakshmi Komarraju, Sathyanath D., Shrikanth Muralidharan

**Affiliations:** 1 Department of Ozone Therapy, National Institute of Naturopathy, Ministry of Ayush, Bapu Bhavan Matoshree, Ramabai Ambedkar Rd, Pune, Maharashtra, India; 2 National Institute of Naturopathy, Ministry of Ayush, Government of India, Pune, India; 3 Department of Clinical Naturopathy, National Institute of Naturopathy, Ministry of Ayush, Government of India, Pune - 411001, India; 4 Department of Research, National Institute of Naturopathy, Ministry of Ayush, Government of India, Pune, India

**Keywords:** Naturopathy, hydrotherapy, vapours bath, Indian medicated vapour bath, shampooing, ayurveda, cultural practices, therapeutic techniques

## Abstract

Naturopathy, a healing science using the natural elements of earth, fire, water and air to treat the disorders, emphasizes the use of water in its solid, liquid and vapor form for the three-fold purpose. Steam or vapor baths, also part of some Indian traditions of Ayurveda and Unani, were introduced into England by Sake Dean Mahomed. Mahomed innovatively adopted Indian Medicated Vapour Baths and shampooing techniques adapting them for European practices, gaining recognition and getting various referrals from hospitals. Thus, his role in bringing Indian cultural and therapeutic practices to the West is significant.

## Background:

A comprehensive understanding of the historical origin and development of Naturopathy in India can only be possible if there is a focus on the personalities and their work in the field. Though the oldest roots of Naturopathy date back to the ancient Vedas, Louis Kuhne from Germany (1835-1901) indeed influenced the movement of Naturopathy during late 19th and early 20th century. Louis Kuhne's experiment on himself leads to the formulation of the doctrine of the "Unity of disease and Unity of Cure," where he describes all diseases as originating from one common cause, the existence of foreign matter (toxin) in the system (body) and emphasizes removal of such matter as the cure [1]. In addition, his suggestion of steam baths for re-establishing skin function offers an extremely practical way of detoxification [2].

Louis Kuhne's book, The New Science of Healing, was first translated in India in the Telugu language by Shri Dronamraju Venkatachalapathi Sharma of West Godavari Andhra Pradesh, in 1886 [3]. By 1897, The New Science of Healing was available in 22 languages worldwide, indicating the wide influence of this work and the great acceptance of Kuhne's principles all over the world [4]. This study will trace the work of Sake Dean Mahomed (1759-1851), almost seven decades before Louis Kuhne, in introducing medicated vapour baths and shampooing from India to England. Therefore, it is of interest to give a comprehensive understanding of the early influences on Naturopathy and the cross-cultural dissemination of therapeutic practices. This study compares and contrasts Mahomed's contributions to Kuhne's, giving insight into global health practices' interconnectivity and the overall development of Naturopathy.

## Mahomed's life in India:

Sake Dean Mahomed was born in 1759 in Patna, which at the time was part of the Bengal Presidency and is now the capital of Bihar, India. His father was working with the 'East India Company. He lost his father when he was almost 11 years old. Following this, Mahomed turned into a camp follower under the wing of Captain Godfrey Baker [5]. Under the patronage of Baker, Mahomed travelled widely in Patna, Buxar, Kanpur, Kolkata, Delhi, Surat and Bombay [6]. He became Subedar but relinquished the rank to follow Captain Baker on his voyage to Europe [7]. In 1784, Sake Dean Mahomed and Captain Baker set sail from Kolkata for England [8]. In his travelogue, The Travels of Dean Mahomet, 1794, Mahomed underlines the existence of public hammams in Surat, India, which have been used for bathing, cupping, massage and sweating. He also underlines the importance of bathing in Indian culture, noting that "even before the age of Pythagoras, the Greeks travelled to India for instruction," illustrating the global influence of Indian thought even in the pre-Hippocratic era [5].

## Mahomed's life in Ireland, London and England:

Sake Dean Mahomed came to Ireland in 1784 with Captain Baker and lived in Cork until 1807. In Cork, he mastered the English language, married Jane Daly in 1786 and wrote his book "The Travels of Dean Mahomet" in 1794. He learned exotic medicine during his stay in Cork, which he practiced professionally, afterwards [10]. Mahomed moved to London in 1807, where he briefly worked with Basil Cochrane at a Bathhouse in Portman Square, introducing therapeutic shampooing. Mahomed and his wife moved to Brighton by 1814 and opened the first shampooing vapor bath, providing treatments for ailments such as rheumatism, gout and stiff joints. To ensure the adaptability of his shampooing and vapour bath methods to the European constitution, Mahomed first tried on himself before offering them to the public [9]. Later, in 1821, they opened Mahomed's Bath, combining Indian medicated vapor baths with herbal remedies. In 1822, Mahomed published Shampooing; or Benefits Resulting from the Use of the Indian Medicated Vapour Bath, showing off the merits of his methods [11]. The book ran through two other editions in 1826 and 1838. Mahomed's Bath finally closed its doors after nearly fifty years of successful business in 1841. Mahomed died on February 24, 1851, at Brighton.

## Mahomed's Methods:

Mahomed practiced shampooing and vapor baths in London and Brighton. The term used by Mahomed, 'shampooing, is borrowed from the Hindi' champi, which represents a head massage. The modern use of a term describing its evolution from just being a head massage to the latest sense of adding chemicals to treat hair [12]. Treatments started with sitting in an herbal vapour bath, then after that, through specially designed sleeves, an assistant performed a full-body therapeutic massage [13]. His wood framework was also painted to seem like bamboo with exotic Indian herbs known and secured from India as included during vaporizing in a chamber. In First Indian Author in English, 1965 Mahomed's son in Michael H. Fisher's work describes his father's method of shampooing by Indian oils involving a series of manipulations, such as friction, extension of ligaments and tendons, squeezing and pounding of the muscles. The hand in lumbago, sciatica and similar conditions was anointed with medicated oils and applied to the affected areas. These techniques are somewhat similar to the Swedish massage techniques applied in Naturopathy in India.

Mahomed's establishment and practices were so successful that nearby hospitals referred patients to him. Michael H Fisher, in his work, First Indian Author in English, 1965 writes, "Mahomed had to issue a notice in the newspaper, cautioning the public not to be confused by imitators," that proves how easily vapour bath houses sprouted up in those days in Brighton. His business thrived and attracted elite clientele; among them are the Kings George IV and William IV of the United Kingdom and Ireland, which patronized Mahomed's Baths and benefited from the treatments. Mahomed was appointed Shampooing Surgeon to the King and awarded a Royal Warrant.

## Conclusion:

Sake Dean Mahomed's pioneering work in introducing Indian therapeutic practices to Britain highlights his role as a key figure in cultural exchange and medical innovation. His adaptation of vapor baths and massage techniques laid the foundation for integrating traditional Indian healing with modern naturopathic therapies. Mahomed's legacy continues to influence global wellness practices, bridging historical traditions with contemporary healthcare.
